# Gamma-irradiated bacille calmette-guÉrin vaccination does not modulate the innate immune response during experimental human endotoxemia

**DOI:** 10.1186/2197-425X-3-S1-A419

**Published:** 2015-10-01

**Authors:** LAC Hamers, M Kox, RJW Arts, B Blok, J Leentjens, MG Netea, P Pickkers

**Affiliations:** Radboud University Medical Center, Intensive Care Medicine, Nijmegen, the Netherlands; Radboud University Medical Center, Internal Medicine, Nijmegen, the Netherlands

## Introduction

Recent insights in sepsis pathology have led to the view that not the initial hyperinflammatory state, but rather a profoundly suppressed state of the immune system, also called immunoparalysis, accounts for the majority of sepsis-related deaths. Therefore, reconstitution of immunocompetence in sepsis is emerging as a promising therapeutic target to improve outcome. Bacille Calmette-Guérin (BCG) vaccine not only protects against tuberculosis, but exerts beneficial effects on other infectious diseases as well. These non-specific effects of BCG seem to be mediated by potentiation of adaptive immunity through heterologous effects, as well as epigenetic functional reprogramming of innate immune cells to an enhanced phenotype, a process described as 'trained immunity', which has been shown *in vitro, ex vivo*, and in animal models. Therefore, BCG-vaccination could represent a novel therapeutic option to treat sepsis-induced immunoparalysis, although its immunomodulatory effects in humans *in vivo* have not yet been investigated. Furthermore, the live BCG vaccine presents a potential risk of disseminated disease in immunoparalyzed patients, which can be circumvented by inactivating the vaccine through gamma-irradiation.

## Objectives

To determine the effects of gamma-irradiated BCG-vaccination on the *in vivo* innate immune responses induced by human endotoxemia. Also, to determine the effects of gamma-irradiated BCG-vaccination on *ex vivo* responsiveness of leukocytes to various inflammatory stimuli.

## Methods

In a randomized double blind placebo-controlled study, healthy male volunteers were vaccinated with gamma-irradiated BCG (n = 10) or placebo (n = 10) and received 1 ng/kg lipopolysaccharide (LPS) intravenously on day 5 after vaccination to assess the *in vivo* immune response. Peripheral blood mononuclear cells were stimulated with various related and unrelated pathogens 5, 8 to 10, and 25 to 35 days after vaccination to assess *ex vivo* immune responses.

## Results

**Figure 1:**
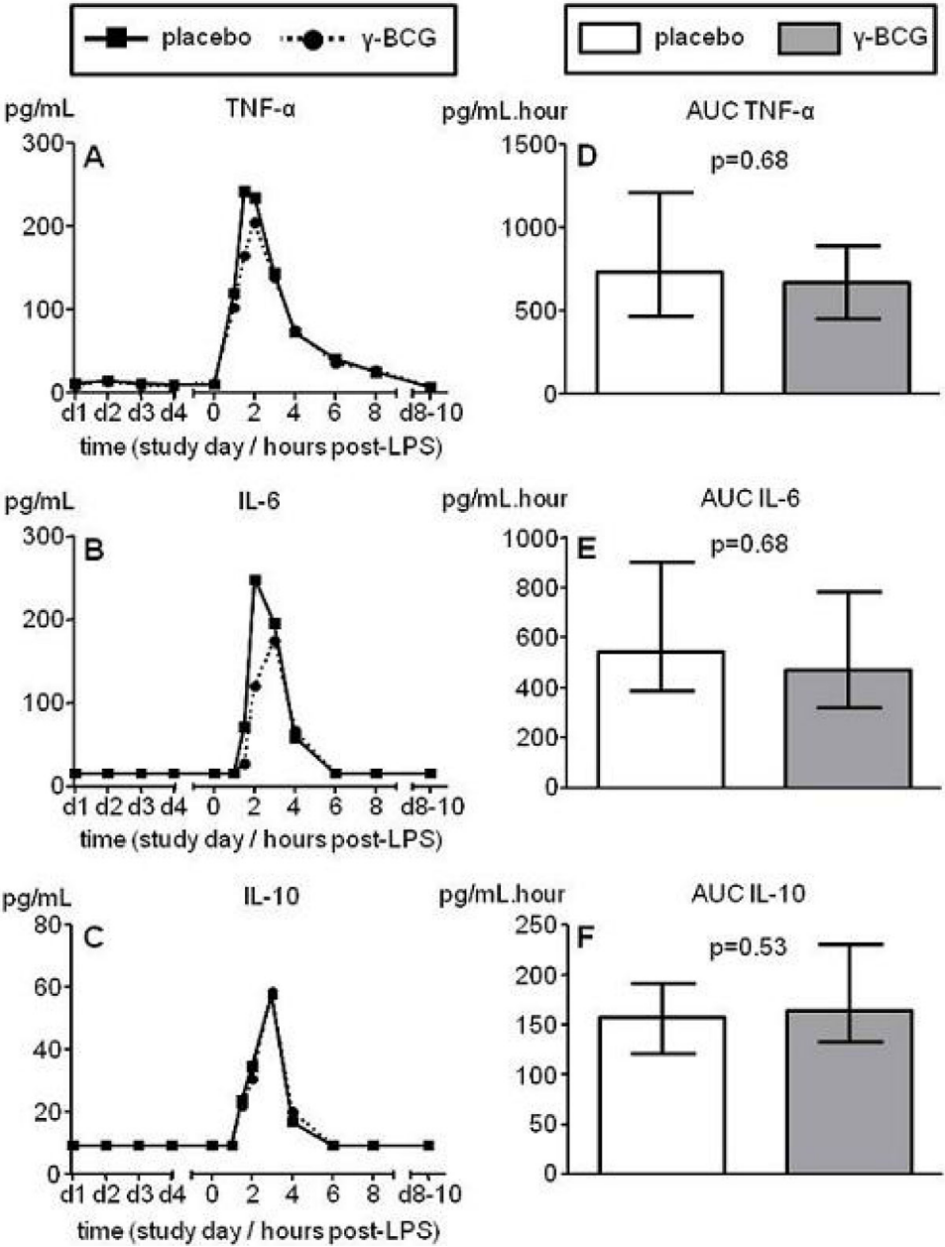
In the panels A and B, median values of pro-inflammatory cytokines TNF-α and Il-6 are depicted while in panel C median values of the anti-inflammatory cytokine IL-10 is shown (n = 10 per group). Panels D-F depict median ± interquartile range of area under curve (AUC) of the respective cytokines (n = 10 per group). P values calculated using Mann-Whitney U-tests.

**Figure 2:**
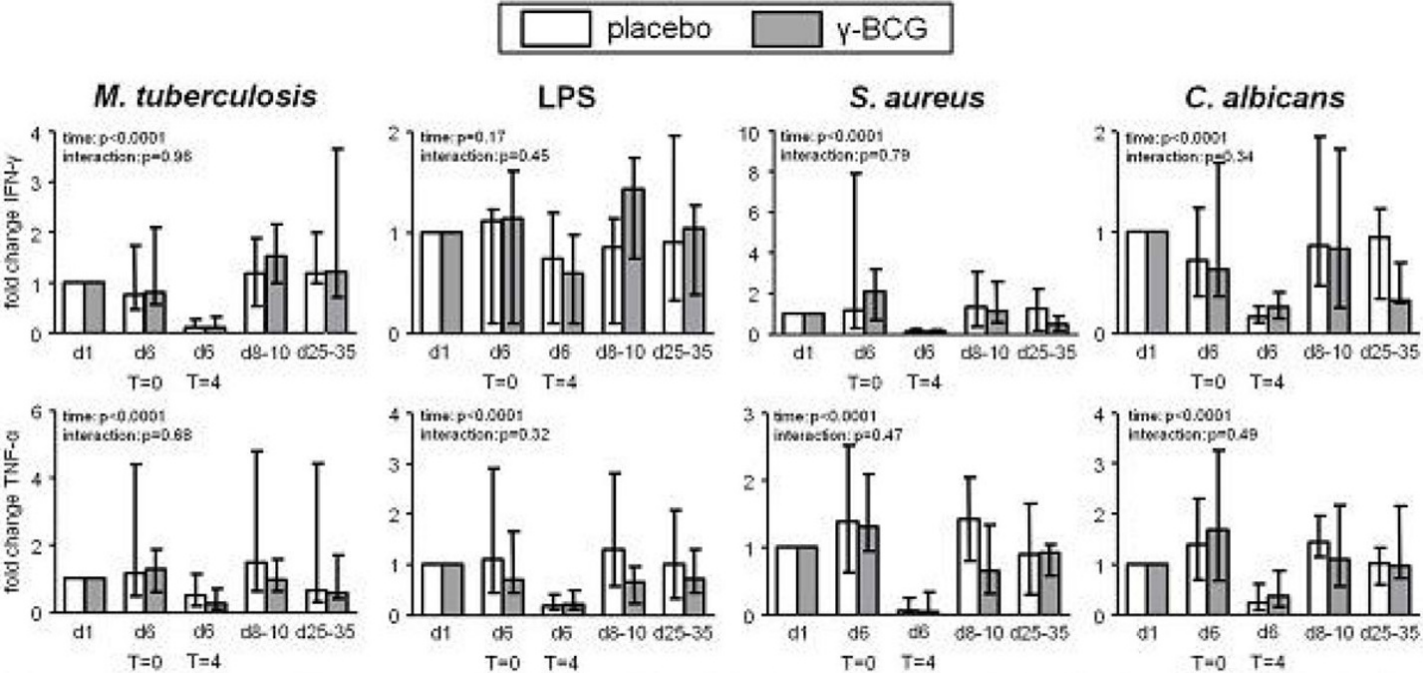
Data expressed as median and interquartile range of the fold change compared with day 1 (before vaccination) (n = 10 per group). p-values calculated using repeated measures two-way analysis of variance (ANOVA, time and interaction terms) on log transformed data. Day 6 was the endotoxemia experiment day.

LPS administration elicited a profound systemic immune response, characterized by increased levels of pro-and anti-inflammatory cytokines, hemodynamic changes, and flu-like symptoms. However, BCG neither modulated this *in vivo* immune response (Figure 1), nor *ex vivo* leukocyte responses at any time-point (Figure 2).

## Conclusions

Gamma-irradiated BCG does not modulate the innate immune response *in vivo* in humans and is therefore unlikely to represent an effective treatment option to restore immunocompetence in patients with sepsis-induced immunoparalysis.

## Grant Acknowledgment

M.G.N. was supported by a Vici grant of the Netherlands Organization for Scientific Research and an ERC Consolidator Grant (#310372).

